# A Preliminary Pilot Study: Metabolomic Analysis of Saliva in Oral Candidiasis

**DOI:** 10.3390/metabo12121294

**Published:** 2022-12-19

**Authors:** Takuya Adachi, Norishige Kawanishi, Narumi Ichigaya, Masahiro Sugimoto, Noriyuki Hoshi, Katsuhiko Kimoto

**Affiliations:** 1 Department of Fixed Prosthodontics, Kanagawa Dental University, Yokosuka 238-8580, Japan; 2 Institute of Medical Sciences, Tokyo Medical University, Shinjuku, Tokyo 160-8402, Japan; 3 Institute for Advanced Biosciences, Keio University, 246-2 Mizukami, Kakuganji, Tsuruoka, Yamagata 997-0052, Japan; 4 Department of Education Planning, Kanagawa Dental University Yokosuka 238-8580, Japan

**Keywords:** oral candidiasis, metabolome analysis, unstimulated saliva, stimulated saliva, *Candida albicans*

## Abstract

Early detection of oral candidiasis is essential. However, most currently available methods are time-consuming and useful only for screening patients. Previous studies on the relationship between oral candidiasis and saliva have focused on saliva volume and not on its components. Therefore, to clarify the effects of oral candidiasis on salivary metabolites, the relationship between salivary components and oral candidiasis was investigated by comparing the salivary metabolites of oral candidiasis patients and those not previously diagnosed with candidiasis. Forty-five participants visiting our university hospital were included and classified into two groups, the *Candida* group and the control group, based on the *Candida* detection test results. The unstimulated saliva was collected using the spitting method over 15 min, and the stimulated saliva was collected using the gum-chewing method over 10 min. The saliva volume was measured, and the saliva samples were frozen and analyzed metabolomically. Metabolome analysis revealed 51 metabolites with peak detection rates exceeding 50%. There was no significant difference in age and sex between the *Candida* and control groups. In the *Candida* group, five metabolites (tyrosine, choline, phosphoenolpyruvate, histidine, and 6-phosphogluconate) were significantly elevated in the unstimulated, two (octanoic acid and uridine monophosphate(UMP)) were significantly increased, and four (ornithine, butyrate, aminovalerate and aminolevulinate) were significantly decreased in the stimulated saliva. This study suggests the possibility of identifying metabolites specific to patients with oral candidiasis, which could aid prompt diagnosis.

## 1. Introduction

Oral candidiasis is a biofilm infection caused by fungi of the genus *Candida* [1]. Pathogenic biofilms cause approximately 80% of cases of human infections [2,3]. Biofilm infections reduce the immune efficacy of antifungal agents and can increase the adverse effects of drug therapy in elderly patients [4]. A decrease in the quantity of saliva and the inappropriate fitting of dentures increase the risk of oral candidiasis [5,6], which further deteriorates the intraoral condition of elderly persons [7,8].

Early detection of oral candidiasis is clinically valuable because this disease is an initial-stage symptom of more serious or life-threatening diseases in the elderly and immunocompromised patients [9]. However, the currently available detection methods of intraoral *Candida* are time-consuming and include examination of smears, swabs, imprint specimens, whole saliva, mouth-rinse liquid, and cell culture [10]. Cell culture-based detection has a low sensitivity [11,12,13]. Moreover, these methods are used only for screening, and the diagnosis depends on the physician’s decision-making. Thus, it is imperative to identify quantitative or objective diagnostic salivary markers of candidiasis.

Saliva serves as a defense against *Candida*. It is composed of substances secreted from multiple sources, including salivary glands and gingival sulcus [14]. Saliva is a complex biological fluid with numerous defense mechanisms, including the formation of a pellicle that protects the teeth and oral mucosa [14]. Salivary components can suppress growth of *Candida* [15]. A decrease in salivary volume and changes in salivary composition are involved in the development of oral candidiasis [16,17,18]. Considering the change in salivary volume as the index, the present authors have reported on the feasibility of alleviating the intraoral symptoms of oral candidiasis [19]. However, the effects of saliva components on the alleviation of oral candidiasis symptoms are unknown. Studies on saliva components have involved proteome, transcriptome, micro-RNA, metabolome, and microbiome analysis [20]. Metabolome or metabolomics is a comprehensive analysis of metabolites [21], and its clinical applications include oral cancer [22], Sjögren’s syndrome [23], pediatric caries [24], periodontal disease [25], and biomarker discoveries of various diseases [16]. The differences in salivary metabolites between the unstimulated and stimulated saliva have also been analyzed [26].

In this study, we hypothesized that there are differences in salivary metabolites between patients with oral candidiasis and healthy controls.

This study aimed to analyze the relationships between oral candidiasis and salivary metabolites using capillary electrophoresis-mass spectrometry (CE-MS), which enables the simultaneous identification and quantification of hundreds of hydrophilic metabolites [27,28,29].

## 2. Materials and Methods

### 2.1. Subjects

Forty-five patients, comprising fifteen men and thirty women, who had visited Kanagawa Dental University Hospital between June 2020 and December 2021, were included. Patients with dementia and/or psychiatric conditions with difficulty in communicating were excluded. Patients with dry mouth were excluded because 1 mL or more was the collection requirement for the test. Smokers were excluded because smoking has been reported to alter salivary composition [30]. Written informed consistent was obtained from all participants. The study was approved by the Ethics Review Committee of Kanagawa Dental University (approval date: 1 June 2016; approval no.: 380).

### 2.2. Study Items

*Candida* detection tests were performed, and saliva was collected at the initial examination.

### 2.3. Candida Detection Tests

At the initial examination, *Candida* was detected using the *Candida* detector, which is based on the Sabouraud agar medium (Kamemizu Chemical Industry Co., Ltd., Osaka, Japan) [31]. Samples were collected by abrasion of the buccal mucosa and tongue surface using a sterilized cotton swab and cultured at 37 °C for 48 h. Participants with test results of 10⁴ colony forming units (CFU)/mL or higher were considered *Candida*-positive and were assigned to the *Candida* group. Participants without the *Candida* infection and those with false-positive results (e.g., 10³ CFU/mL) were assigned to the control group [19]. Although the *Candida* detector is relatively easy to use, it cannot identify *Candida* species.

### 2.4. Saliva Collection

Samples of the unstimulated and stimulated saliva, whole saliva in both cases, were collected from the participants.

The unstimulated saliva was collected by the spitting method. Swallowing of saliva was prohibited during the saliva collection period. Before the start of the collection period, the subjects swallowed the saliva present in their oral cavities, after which saliva was collected by allowing it to drip into a sterilized Falcon tube (Corning, Inc., Tokyo, Japan) over 15 min [32,33,34].

The stimulated saliva was collected by the gum-chewing method. The subjects were instructed to chew Freezone gum (Lotte Co., Ltd., Tokyo, Japan) and to not swallow saliva during the collection period. Saliva was collected by allowing it to drip into a sterilized Falcon tube for 10 min [34,35,36].

The same physicians collected both the stimulated and unstimulated saliva. To minimize variations in saliva, the participants were instructed not to eat or drink anything, except water, for 2 h before collection. In addition, the saliva samples were collected on a Monday or Tuesday morning (between 9:00 AM and 11:00 AM) to avoid the bias of diurnal variation in saliva composition [34].

### 2.5. Metabolome Analysis

In the present study, metabolome analysis was performed in accordance with the previously described methods [30].

Briefly, frozen saliva was thawed, passed through a 5-kDa cut-off filter (Nihon Pall, Ltd., Tokyo, Japan), centrifuged for at least 2.5 h at 9100× *g* and 40 °C, and filtered to remove high-molecular-weight compounds. Next, 5 μL of Milli-Q water (Millipore Corporation, Bedford, MA, USA) containing methionine sulfone, 2-[N-morpholino]-ethanesulfonic acid, D-camphor-10-sulfonic acid, 3-aminopyrrolidine, and trimesate, each at 2 mmol/L, was added to 45 μL of filtrate, followed by mixing. The processed samples were analyzed by capillary electrophoresis-time-of-flight-mass spectrometry (CE-TOFMS) in positive and negative modes. The following procedure was used to identify metabolites. CE-MS data were analyzed using MasterHands (Keio University) with noise filtering, subtraction of baselines, peak integration for each sliced electropherogram, estimation of accurate m/z in MS, alignment of multiple datasets to generate peak matrices, and identification of each peak by matching m/z values and corrected migration times to corresponding entries in a standard library. Metabolite concentrations in CE-MS were calculated based on the ratio of peak area divided by the area of the internal standards in the samples and standard compound mixtures. Polyamine LC-MS data were used for subsequent analyses because both methods redundantly detected their peaks.

### 2.6. Data Analysis

Data analysis was performed as described previously [26,34]. For metabolite data analysis, MasterHands software (version 2.18.0.2; Keio University, Yamagata, Japan) was used. Peaks were detected as far as possible, and the noise was eliminated. For the identification of metabolites, the *m*/*z* value and normalized transition time were compared with those of standard compounds. For measuring the concentration of each metabolite, internal and external standards were used.

Metabolome profile diversity was determined overall by principal component analysis (PCA). The χ^²^ test was used to assess sex differences in oral candidiasis. The Mann–Whitney U-test was used to compare salivary metabolites between the control and *Candida* groups, with the significance level taken to be 0.05. The Shapiro–Wilk test was used to test normality. Mann–Whitney tests were used to compare quantitative values for two group comparisons. *p*-values were corrected by the false discovery rate (Benjamini–Hochberg) method to consider the multiple independent tests. The following software was used for the visualization and analyses: Mev TM4 (ver. 4.9.0, http://mev.tm4.org/) (accessed on 1 August 2022), MetaboAnalyst (ver. 5.0, https://www.metaboanalyst.ca/) (accessed on 1 August 2022), and GraphPad Prism (ver. 5.0.2, GraphPad Software, Inc., San Diego, CA, USA).

## 3. Results

### 3.1. Subjects

There were 20 participants in the control group (7 men, 13 women) and 25 in the *Candida* group (8 men, 17 women). No significant sex differences were found between the control and *Candida* groups by the χ^²^ test (*p* = 1.0, with the significance level taken to be 0.05 or lower). The mean (± standard deviation) ages were 76.2 ± 6.9 years in the control group, and 76.6 ± 6.4 years in the *Candida* group (Table 1).

### 3.2. Overview of the Detected Salivary Metabolites

**Figure 1:**
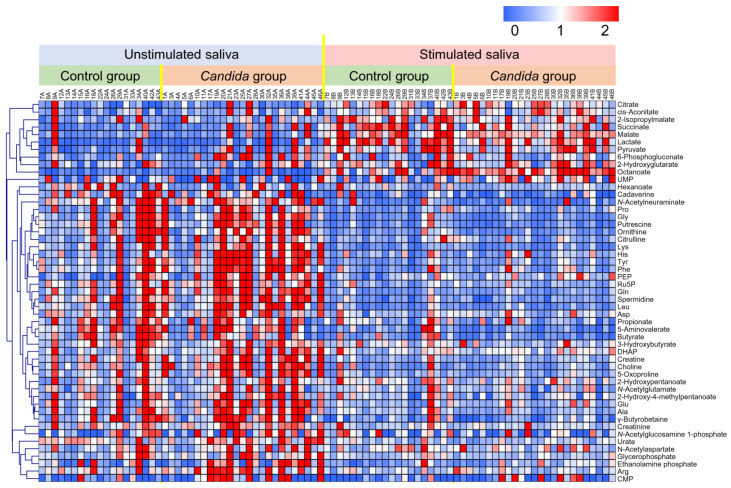
Heat map of metabolites detected in participants’ unstimulated and stimulated saliva. The 51 metabolites frequently detected in >50% of all samples are visualized. The concentration of each metabolite is divided by its average concentration. Clustering was conducted based on Pearson correlation. Metabolites with low and high concentrations are shown in blue and red, respectively.

A total of 510 metabolites were identified and quantified (Table S1). Of these, 51 frequently detected metabolites (>50% of samples) were visualized as a heat map (Figure 1). In this heat map, samples showing similar salivary metabolite concentration patterns were closely aligned. Lower and higher concentrations are indicated by blue and red, respectively. Remarkable differences between the unstimulated and stimulated saliva were observed. In addition, the metabolites clustered at the top of the heat map, from citrate to octanoate, tended to be detected at higher concentrations in the stimulated than in the unstimulated saliva. Comparison between the control and *Candida* groups showed that metabolites in the unstimulated saliva were detected overall at higher concentrations in the *Candida* group. In contrast, in the stimulated saliva, the concentrations of metabolites were widely scattered, and numerous metabolites were detected at low concentrations in the *Candida* group.

### 3.3. Principal Component Analysis (PCA)

**Figure 2:**
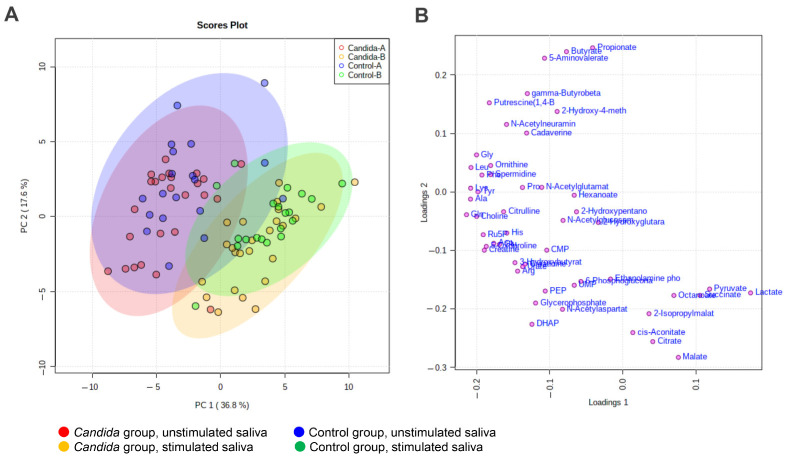
Prinicipal Component Analysis (PCA) of metabolites detected in participants’ unstimulated and stimulated saliva. PCA of the detected salivary metabolites was performed. Score plot and loading plot of the PCA with the 51 metabolites were used for visualization. (**A**) Score plot of metabolites detected in participants’ unstimulated and stimulated saliva. X and Y-axes indicate the contribution ratios of PC1 and PC2, respectively. Each point on the score plot represents one saliva sample, and the shorter the distances between points the higher similarity of their salivary metabolite concentration patterns. (**B**) Loading plot of metabolites detected in participants’ saliva. In the loading plot, each point represents one metabolite. The used options to process the data were (1) sample normalization: normalization by sum, (2) data transformation: Log transformation, and (3) data scaling: Auto scaling.

Score plots from the PCA of salivary metabolome profiles are shown for the unstimulated and stimulated saliva. The contribution ratios of the first and second principal components (PC1 and PC2) were 36.8% and 17.6%, respectively (Figure 2A). In the control and *Candida* groups, the unstimulated and stimulated saliva are represented by different colors on the score plots, as follows: the unstimulated saliva in control group: blue; the stimulated saliva in control group: green; the unstimulated saliva in *Candida* group: red; and the stimulated saliva in *Candida* group: yellow. The contribution ratio of PC1 was 36.8%, higher than that of PC2 (17.6%). Numerous metabolites with profiles similar to PC1 were found. PC1 made a greater contribution in the unstimulated saliva than in the stimulated saliva, showing that numerous metabolites vary in connection with the saliva volume and the saliva components. In addition, both the unstimulated and stimulated saliva were compared between the *Candida* and control groups, and the trend plotted on the lower left region in Figure 2A was found; a comparison of the control and *Candida* groups showed that in the *Candida* group, the distribution tended to be in the lower region (PC2 < 0; Figure 2A).

In the loading plot, one point represents one metabolite. All except eight of the metabolites are shown in the left half of the graph (PC1 < 0) showing that PC1 negatively correlates with the overall metabolites. PC2 showed a wide scatter between metabolites, suggesting that PC2 may be responsible for participants’ unique metabolite profiles (Figure 2B).

### 3.4. Comparison with Volcano Plot

**Figure 3:**
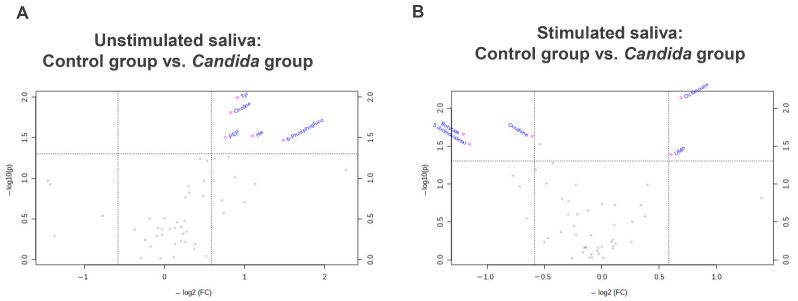
Volcano analysis of metabolites. (**A**) unstimulated saliva. (**B**) stimulated saliva. X-axes indicate the log_2_ of fole change (*Candida* group/control group) of the averaged concentration. The metabolites above the horizontal lines (Y = 1.3, i.e., *p* = 0.05) indicate the significantly different between two groups. Mann–Whitney test was used to calculate the *p*-values.

After plotting the 51 metabolites, the unstimulated and stimulated saliva of the control and *Candida* groups were compared, as shown in Figure 3A,B. In the unstimulated saliva, five metabolites were higher in the *Candida* group than in the control group (tyrosine, choline, phosphoenolpyruvate, histidine, and 6-phosphogluconate; Figure 3A), whereas in the stimulated saliva, two metabolites were higher in the *Candida* group [octanoate and uridine monophosphate (UMP)], and three were lower in the *Candida* group [ornithine, butyrate, and 5-aminopentanoate (aminovalerate); Figure 3B].

### 3.5. Comparison between Control and Candida Groups in Connection with Oral Candidiasis

**Figure 4:**
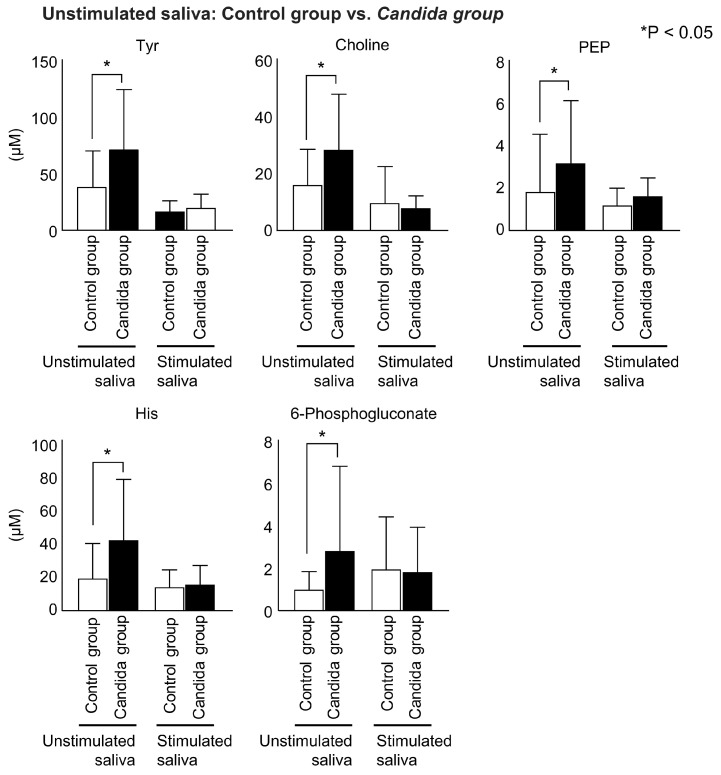
Inter-group comparison of metabolites detected in participants’ unstimulated saliva. Salivary metabolites in the *Candida* group and control group were compared using the Mann–Whitney test. Significant differences were found between five metabolites in unstimulated saliva.

The metabolites that showed significant differences in the unstimulated and stimulated saliva between the control and *Candida* groups are shown. In the unstimulated saliva, tyrosine, choline, phosphoenolpyruvate, histidine, and 6-phosphogluconate showed significantly higher concentrations in the *Candida* group than in the control group (Figure 4).

**Figure 5:**
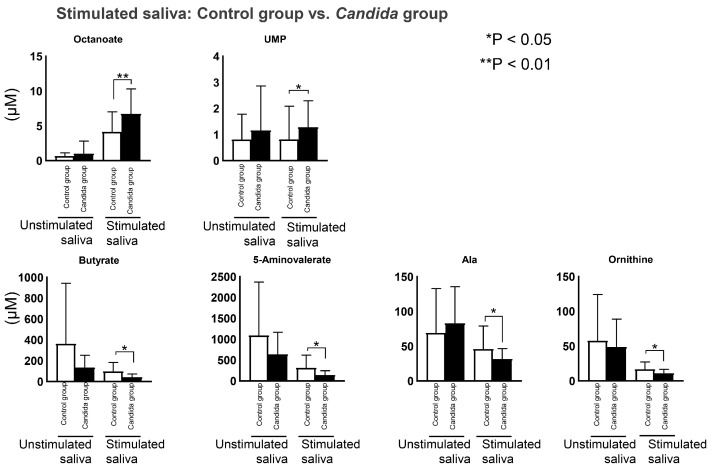
Inter-group comparison of metabolites detected in participants’ stimulated saliva. Salivary metabolites in the *Candida* group and control group were compared using the Mann–Whitney test. In stimulated saliva, four metabolites showed significantly lower concentration, and two metabolites showed significantly higher concentration.

In the stimulated saliva, two metabolites were at significantly higher concentrations in the *Candida* group, including octanoate and UMP, and four metabolites were at significantly lower concentrations, these being ornithine, butyrate, aminovalerate, and aminolevulinate (Figure 5).

## 4. Discussion

In this study, as hypothesized, we found significant differences in several salivary metabolites between patients with oral candidiasis and those not previously diagnosed with candidiasis. Our metabolome analysis of salivary metabolites of 45 participants was performed using the *Candida* detector. The results were compared between oral candidiasis patients (the *Candida* group) and healthy people (the control group). In the *Candida* group, five metabolites in the unstimulated saliva were significantly higher than in the control group. In the stimulated saliva, four metabolites were at significantly lower levels, and two metabolites were at significantly higher levels than in the control group.

The saliva metabolome analysis used in this study has several advantages. The risk of acquired infection is low because saliva can be collected noninvasively. In addition, the simplicity of the equipment allows saliva to be collected even in clinically difficult situations, and this analysis method can be used as a highly cost-effective approach to mass screening [37]. Including saliva metabolome analysis in the primary examination could greatly reduce the burden on medical departments, such as the need for expensive equipment and invasive test methods [38,39,40]. In addition, the burden imposed by sample collection is minor, and thus, sampling can be performed multiple times. It also has potential for effective monitoring of the disease [41,42]. Four methods of saliva collection are commonly used: (i) collection without stimulation (unstimulated saliva); (ii) collection with stimulation by chewing gum (stimulated saliva); (iii) collection of saliva from the parotid gland by acid stimulation, using a Lashley cup; and (iv) collection of saliva from cotton swabs [43]. However, only the first two methods were used in the present study. Differences between metabolites in the unstimulated and stimulated saliva have been reported in previous studies [26] and some metabolites may potentially be detected in the stimulated saliva despite not occurring at a significant level in the unstimulated saliva. Therefore, analysis of metabolites in the stimulated saliva can be advantageous to identify salivary metabolites associated with oral candidiasis [26]. The unstimulated saliva is used in many salivary metabolome studies, but the stimulated saliva was also used in this study. The compositions of the stimulated and unstimulated saliva are different, and microbiological studies have reported that the stimulated saliva along with the unstimulated saliva are suitable specimens [44]. Analysis of both the unstimulated and stimulated saliva is diagnostically advantageous. The present findings indicate that, on comparing the control and *Candida* groups, metabolites showed significant differences in both the unstimulated and stimulated saliva.

Metabolites showing significant differences were found in the *Candida* group. In the unstimulated saliva, tyrosine, choline, phosphoenolpyruvate, histidine, and 6-phosphogluconate were elevated. Tyrosine was detected at a high concentration due to the *Candida albicans* extracellular metabolome profile [45]. Tyrosine is converted to tyrosol by decarboxylation and deamination reactions, but tyrosol induces *Candida albicans* filament formation and promotes biofilm formation [46]. *Candida albicans* is pleomorphic and undergoes morphological changes in accordance with changes in the host environment, taking yeast-type, pseudo-mycelial, and true mycelial forms [47], indicating that metabolites play important roles in this morphogenesis [48]. In addition, histidine is involved in the over-expression of pyrimidine synthase (THI5p) by *Candida albicans* [49], and the concentrations of octanoate and UMP, two salivary metabolites, have been found to increase in the stimulated saliva. Octanoate is a metabolite common to the following three *Candida* species: *C. albicans*, *C*. *glabrata*, and *C. tropicalis* [50]. In addition, UMP is involved in nucleic acid metabolism, and it has previously been detected at high concentrations in *Candida* strains isolated from respiratory organs [45]. UMP has been reported to be connected to *Candida* pathogenicity [51]. Four salivary metabolites, including ornithine, butyrate, aminovalerate, and aminolevulinate, showed decreased levels. Ornithine and butyrate have been reported to be associated with periodontal disease [52,53,54]. Ornithine has been put forward as a potential marker for inflammation in periodontal disease [52], and butyrate is produced by anaerobic, Gram-negative bacteria, which are periodontal disease pathogens [48,54]. In the present study, metabolites associated with periodontal disease were decreased in the *Candida* group. Previous reports have linked periodontal disease pathogens and *Candida albicans* [55]. Increased cell density of *Porphyromonas gingivalis* has been reported to decrease the number of viable yeast cells and inhibit *Candida* biofilm formation [55]. Therefore, the decrease in periodontal disease-related metabolites may have promoted an increase in the number of *Candida* yeast cells and biofilm formation. Oral candidiasis is closely associated with pathogens that cause an opportunistic infection [56]. In this study, effects of pathogens other than *Candida* were not investigated. However, the present results and previous studies suggest an association between *Candida* and periodontal pathogens. In addition, tyrosol, UMP, among other metabolites, are associated with *Candida* biofilm formation and pathogenicity [46,51]. The described methods may help not only to verify the presence of *Candida* but also to determine its pathogenicity.

This study had several limitations:

1. Whole-mouth saliva was used, including components from serum, salivary gland secretions, gingival sulcus exudates, mucosal exudates, and intraoral microorganisms [14]. Bacterial metabolites, in particular, have major effects, but in this study, periodontal disease, caries, and other oral diseases were not considered [57].
2. The participants’ systemic diseases or medication status were not considered. The subjects in both the control and *Candida* groups had mean ages over 70 years. In an elderly population, it is difficult to sample subjects who have no systemic diseases and are not currently taking medication. To verify the generalizability of our findings, a large scale study is necessary with more detailed analysis.
3. Constructing and unifying a database by including other metabolome profiles would enable more accurate diagnosis.
4. *Candida* species were not identified in this study. The pathogens responsible for candidiasis include *C. albicans, C. glabrata, C. parapsilosis, C. tropicalis, and C. krusei*. It is essential to evaluate the effects of different species on the salivary metabolites.
5. The target age group for this study was 60 years and older. This was established to avoid bias because previous studies have shown that salivary metabolites change with age [58]. However, since there are also young patients with oral candidiasis, analysis of a wide range of age groups is an issue for future research.

## 5. Conclusions

This study analyzed salivary metabolites having a significant association with oral candidiasis. The results suggests that it will be possible to identify biomarkers specific to oral candidiasis among salivary metabolites. The results may contribute to the early detection of oral candidiasis and the establishment of new treatment methods and may deepen our knowledge of oral candidiasis and salivary metabolites.

## Figures and Tables

**Table 1:** Patient characteristics.

	Control Group	Candida Group
Age (mean ± SD)	76.2 ± 6.9	76.6 ± 6.4
Sex (men/women)	7/13	8/17

## Data Availability

All the raw data elaborated in this study are provided in Table S1.

## Supplementary Materials

The following supporting information can be downloaded at: https://www.mdpi.com/article/10.3390/metabo12121294/s1, Table S1: Sample concentrations of salivary metabolites for all patients: It is the concentration of a sample of salivary metabolites in all patients. The 51 metabolites frequently detected from these (>50% of samples) were visualized as a heat map (Figure 1).
